# Oligomeric States and Hydrodynamic Properties of Lysyl Oxidase-Like 2

**DOI:** 10.3390/biom11121846

**Published:** 2021-12-08

**Authors:** Alex A. Meier, Hee-Jung Moon, Ronald Toth, Ewa Folta-Stogniew, Krzysztof Kuczera, C. Russell Middaugh, Minae Mure

**Affiliations:** 1 Department of Chemistry, University of Kansas, Lawrence, KS 66045, USA; aameier@ku.edu (A.A.M.); hjmoon@ku.edu (H.-J.M.); kkuczera@ku.edu (K.K.); 2 Department of Pharmaceutical Chemistry, School of Pharmacy, University of Kansas, Lawrence, KS 66047, USA; ronald.toth@gmail.com (R.T.IV); middaugh@ku.edu (C.R.M.); 3 W.M. Keck Biotechnology Resource Laboratory, Department of Molecular Biophysics and Biochemistry, Yale School of Medicine, New Haven, CT 06511, USA; ewa.folta-stogniew@yale.edu; 4 Department of Molecular Biosciences, University of Kansas, Lawrence, KS 66045, USA

**Keywords:** lysyl oxidase-like 2, scavenger receptor cysteine-rich, extracellular matrix, hydrodynamic radius, analytical ultracentrifugation, isoelectric points

## Abstract

Lysyl oxidase-like 2 (LOXL2) has emerged as a promising therapeutic target against metastatic/invasive tumors and organ and tissue fibrosis. LOXL2 catalyzes the oxidative deamination of lysine and hydroxylysine residues in extracellular matrix (ECM) proteins to promote crosslinking of these proteins, and thereby plays a major role in ECM remodeling. LOXL2 secretes as 100-kDa full-length protein (fl-LOXL2) and then undergoes proteolytic cleavage of the first two scavenger receptor cysteine-rich (SRCR) domains to yield 60-kDa protein (Δ1-2SRCR-LOXL2). This processing does not affect the amine oxidase activity of LOXL2 in vitro. However, the physiological importance of this cleavage still remains elusive. In this study, we focused on characterization of biophysical properties of fl- and Δ1-2SRCR-LOXL2s (e.g., oligomeric states, molecular weights, and hydrodynamic radii in solution) to gain insight into the structural role of the first two SRCR domains. Our study reveals that fl-LOXL2 exists predominantly as monomer but also dimer to the lesser extent when its concentration is <~1 mM. The hydrodynamic radius (*R*_h_) determined by multi-angle light scattering coupled with size exclusion chromatography (SEC-MALS) indicates that fl-LOXL2 is a moderately asymmetric protein. In contrast, Δ1-2SRCR-LOXL2 exists solely as monomer and its *R*_h_ is in good agreement with the predicted value. The *R*_h_ values calculated from a 3D modeled structure of fl-LOXL2 and the crystal structure of the precursor Δ1-2SRCR-LOXL2 are within a reasonable margin of error of the values determined by SEC-MALS for fl- and Δ1-2SRCR-LOXL2s in mature forms in this study. Based on superimposition of the 3D model and the crystal structure of Δ1-2SRCR-LOXL2 (PDB:5ZE3), we propose a configuration of fl-LOXL2 that explains the difference observed in *R*_h_ between fl- and Δ1-2SRCR-LOXL2s in solution.

## 1. Introduction

Lysyl oxidase-like 2 (LOXL2) is a member of the LOX-family of proteins that are lysine tyrosylquinone (LTQ)- and copper (II)- dependent amine oxidases. The LTQ cofactor is post-translationally derived from the conserved Tyr and Lys residues in the active site [1,2]. LOXL2 oxidatively deaminates lysine and hydroxylysine residues in extracellular matrix (ECM) proteins such as tropoelastin and collagen type IV to initiate their crosslinking and promote ECM remodeling [3,4,5]. In addition to the traditional ECM substrates, platelet growth factor receptor β (PDGFRβ) was recently identified as a cell-surface substrate of LOXL2 [6]. Elevated expression of LOXL2 has been associated with poor prognosis in metastatic/invasive cancers and fibrotic disorders [7,8,9]. Small molecule inhibitors and inhibitory RNAs have shown to effectively retard disease progression in both in vitro and in vivo studies [6,9,10,11,12,13,14,15,16]. Despite its prominence as a therapeutic target, little progress has been made on structure-based drug design for LOXL2 specific inhibitors because of the lack of 3D structural information of the catalytically competent (mature) form of LOXL2.

**Figure 1:**
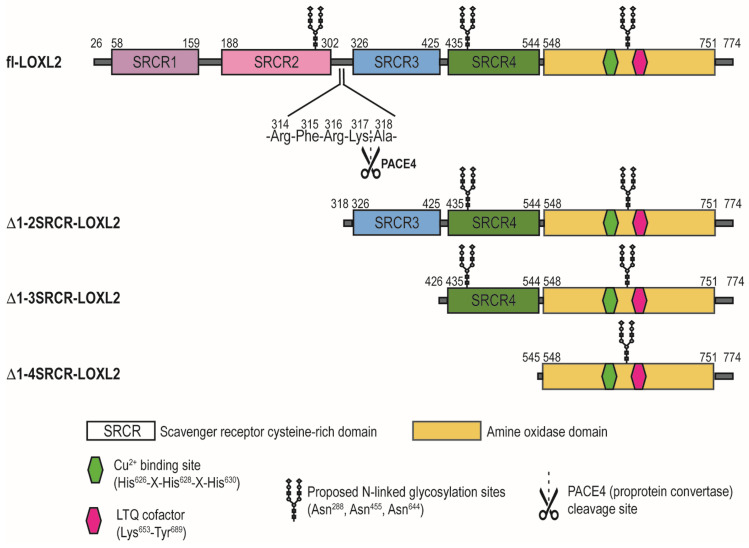
A schematic diagram of rLOXL2s in this study. fl-LOXL2: full-length LOXL2; Δ1-2SRCR-LOXL2: LOXL2 which lacks the first two SRCR domains; Δ1-3SRCR-LOXL2: LOXL2 which lacks the first three SRCR domains; Δ1-4SRCR-LOXL2: LOXL2 which lacks all four SRCR domains.

The *loxl2* gene encodes four scavenger receptor cysteine-rich (SRCR) domains at the N-terminus and the amine oxidase domain at the C-terminus (Figure 1). The C-terminal amine oxidase domain is conserved among the LOX-family of proteins and the catalytic domains of LOXL2 and LOX share 68% similarity and 49% identity. SRCR domains are ancient motifs found on the cell surface and are involved in protein–protein, protein-substrate or receptor-ligand interactions [17,18]. SRCR domains are 90–110 amino acids long and contain highly conserved Cys residues. Based on the numbers of Cys residues and intradomain disulfide bonds, SRCR domains are classified into two categories (type A and type B). Type A domain is characterized by six Cys residues (three disulfide bonds) while type B domain is characterized by eight Cys residues (four disulfide bonds). The SRCR domains of LOXL2 contain six Cys residues per domain, therefore it is categorized as type A [17]. The physiological role of SRCR domains of LOXL2 largely remains elusive.

**Figure 2:**
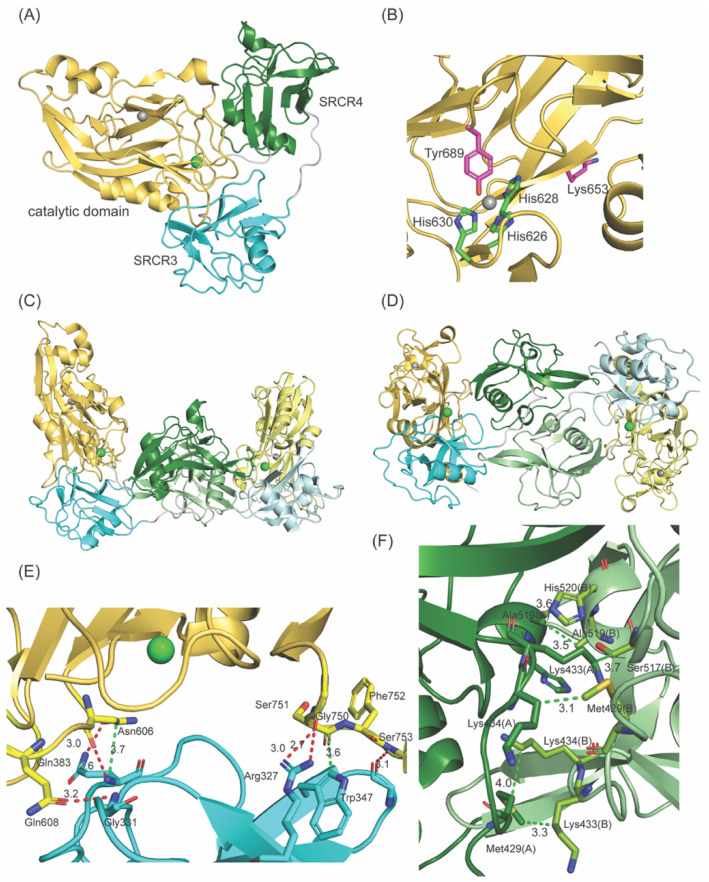
X-ray structure of Δ1-2SRCR-LOXL2 in the precursor form (PDB: 5ZE3). (**A**) Monomer (**B**) The active site structure. The precursor residues (Lys653, Tyr689) are 16.6 Å apart. Zn^2+^ (gray sphere) occupies the predicted Cu^2+^ binding site (His626-X-His628-X-His630). Ca^2+^ is shown as a green sphere. (**C**) In each asymmetric unit (ASU), two molecules are observed. The intermolecular association between monomers is mediated mainly through the 4th SRCR domains (in forest and light green). (**D**) A top view of the ASU shown in (**C**). (**E**) The 3rd SRCR domain (in cyan) interacts with the catalytic domain (in yellow) mostly through hydrogen bonding interactions (in red) but also through van der Waals interactions (in green). (**F**) The intermolecular association observed in between the 4th SRCR domains (shown in **C**,**D**) are all through van der Waals interactions (in green). Numbers in (**A**,**E**,**F**) are all in Å.

LOXL2 secretes as ~100-kDa full-length protein (fl-LOXL2) and that undergoes proteolytic cleavage of the first two SRCR domains by PACE4, a proprotein convertase, at Arg^314^-Phe^315^-Arg^316^-Lys^317^↓Ala^318^ (underlined: the recognition sequence of PACE4) to yield ~60-kDa protein (Δ1-2SRCR-LOXL2) [4]. In vitro, this processing does not affect the amine oxidase activity of LOXL2 in the oxidation of cadaverine and tropoelastin, but increases the solubility of LOXL2 [4]. The physiological significance of this processing is still unclear. In 2018, a 2.4 Å crystal structure of a Zn^2+^-bound precursor form (lacking the LTQ cofactor) of Δ1-2SRCR-LOXL2 became available [19]. The overall structure of Δ1-2SRCR-LOXL2 is in a triangular shape (Figure 2A) where the 3rd SRCR domain interacts with the catalytic domain through in total of six hydrogen bonds and two van der Waals interactions (Figure 2E). The 4th SRCR domain has no interaction with either the catalytic domain or the 3^rd^ SRCR domain (Figure 2C,D). The precursor residues of LTQ (Lys653 and Tyr689) were 16.6 Å apart (Figure 2B) and it was suggested that a substantial conformational change is required to enable the LTQ cofactor formation.

In this study, we focused on understanding the biophysical significance of the proteolytic cleavage of the first two SRCR domains using catalytically competent forms (containing the LTQ cofactor and Cu^2+^) of full-length (fl-LOXL2) and 60-kDa form (Δ1-2SRCR-LOXL2) of LOXL2. The latter was generated from K317R mutant (furin-cleavage site, ^315^Arg-Phe-^316^Arg-^317^Arg, was engineered) of LOXL2. We determined the oligomeric states and hydrodynamic radii (*R*_h_) of the two forms of LOXL2. The latter values are compared with those calculated from a 3D model structure of the precursor fl-LOXL2 being generated by AlphaFold V2 [20] and the crystal structure of the precursor Δ1-2SRCR-LOXL2 [19], respectively.

## 2. Materials and Methods

### 2.1. Protein Purification

The fl- and Δ1-2SRCR-LOXL2s were both produced in FreeStyle™ 293 Expression System (Thermo Fisher Scientific, Lenexa, KS, USA) and were purified as described previously [4]. Δ1-3SRCR- and Δ1-4SRCR-LOXL2s were produced in DES^®^ system (Thermo Fisher Scientific) and were purified as described previously [21].

### 2.2. PAGE Analysis

For SDS-PAGE, 4 μg of protein samples was loaded on to a 10% TGX™ FastCast™ (Bio-Rad Laboratories, Hercules, CA, USA) polyacrylamide gel. Protein samples were prepared with 4X sample loading buffer (Bio-Rad Laboratories) for reducing conditions and with Pierce™ LDS sample buffer, non-reducing (4X) (Thermo Fisher Scientific) for non-reducing conditions. For native PAGE, protein samples were loaded on to a NativePAGE™ 4 to 16% Bis-Tris Mini Protein Gels (Thermo Fisher Scientific) with NativeMark™ Unstained Protein Standard (Thermo Fisher Scientific) and separated at 125 V for 2 h. A recombinant prolyl-4-hydroxylase (homodimer, 49.2-kDa) was used as the protein standard [22].

### 2.3. SEC-MALS

The light scattering data were collected from size exclusion chromatography (SEC) using a Superdex 200 10/300, HR Size Exclusion Chromatography column (GE Healthcare, Piscataway, NJ, USA) connected to Agilent 1200 High Performance Liquid Chromatography (HPLC) System (Agilent Technologies, Wilmington, DE, USA) equipped with an autosampler. The elution from SEC was monitored by a photodiode array (PDA) UV/VIS detector (Agilent Technologies, Wilmington, DE, USA), differential refractometer (OPTI-LabrEx Wyatt Corp., Santa Barbara, CA, USA), static and dynamic, multiangle laser light scattering (LS) detector (HELEOS II with QELS capability, Wyatt Corp., Santa Barbara, CA, USA). The SEC-UV/LS/RI system was equilibrated in 50 mM Tris, pH 8.0 containing 150 mM NaCl and 0.01% sodium azide at a flowrate of 0.5 mL/min or 1.0 mL/min. Two software packages were used for data collection and analysis: the Chemstation software (Agilent Technologies, Wilmington, DE, USA) controlled the HPLC operation and data collection from the multi-wavelength UV/VIS detector, while the ASTRA software (Wyatt Corp., Santa Barbara, CA, USA) collected data from the refractive index detector, the light scattering detectors, and recorded the UV trace at 280 nm sent from the PDA detector. The weight average molecular masses, Mw, were determined across the entire elution profile in the intervals of 1 s from static LS measurement using ASTRA software as previously described [23]. Hydrodynamic radii, *R*_h_, were measured from an “on-line” dynamic LS measurement every 2 s. The dynamic light scattering signal was analyzed by the method of cumulants [24].

### 2.4. Analytical Ultracentrifugation

Analytical ultracentrifugation (AUC) experiments were performed on an Optima XL-I (Beckman Coulter, Fullerton, CA, USA) analytical ultracentrifuge equipped with a scanning UV-Visible optical system set at 280 nm and an An-60 Ti 4-hole rotor. Sedimentation velocity experiments were conducted using a Beckman double-sector Epon centerpiece cell with 12 mm sapphire windows at 40,000 RPM. The experiments were performed in 50 mM HEPES (pH 8.0) containing between 0.088 mg/mL and 0.88 mg/mL fl-LOXL2. The data were analyzed using the continuous sedimentation coefficient distribution model *c*(*s*) of SEDFIT [25]. Sedimentation equilibrium experiments were performed using a six-sector Epon centerpiece cell with 12 mm sapphire windows (Beckman Coulter). Rotor speeds of 10,000, 12,500, and 15,000 RPM were applied. The data were analyzed using HeteroAnalysis to fit molecular weight, n, ln(*K*) and baseline [26].

### 2.5. pI Determination of LOXL2s

Purified recombinant LOXL2s were subjected to 2-dimensional (2D) gel electrophoresis. ReadyStrip™ IPG Strips (11 cm pH 3–10 nonlinear, immobilized pH gradient, IPG strip) (Bio-Rad Laboratories) were rehydrated with 200 μL of rehydration buffer [8 M urea, 2% CHAPS, 50 mM dithiothreitol (DTT), 0.2% Bio-Lyte^®^ 3/10 ampholyte, and 0.001% Bromophenol Blue] containing 100 μg of protein for 12 h in a rehydration tray overlayed with 5 mL of mineral oil. The excess mineral oil was removed from the rehydrated strips by blotting the strip on a filter paper. The IPG strips were transferred to the running tray of a PROTEAN^®^ i12™ IEF system (Bio-Rad Laboratories) before being overlaid with another 5 mL of mineral oil and run using the recommended procedure for 11 cm pH 3–10 IPG strips (e.g., 250 volts for 20 min, 8000 volts for 1 h, and 8000 volts for 26,000 volt hours, with a total run time of approximately 6 h at 20 °C). The IPG strips were taken out of the IEF system, and the excess mineral oil was removed by blotting. The strips were then equilibrated with 4 mL of SDS-PAGE Equilibration Buffer 1 (6 M urea, 2% SDS, 0.375 M Tris-HCl (pH 8.8), 20% glycerol and 2% (*w*/*v*) DTT) for 15 min and SDS-PAGE Equilibration Buffer 2 [6 M urea, 2% SDS, 0.375 M Tris-HCl (pH 8.8), 20% glycerol, and 0.135 M iodoacetamide] from the ReadyPrep^TM^ 2D Starter Kit (Bio-Rad Laboratories) for 15 min. Equilibrated IPG strips were rinsed in 1X TGS running buffer before mounting onto an AnykD™ Criterion™ TGX Stain-Free™ Precast Gel (Bio-Rad Laboratories) and overlaying with 0.5% agarose. The 2D gel was run at 200 V for 60 min or until the dye front reached the bottom of the gel. The gels were then stained using Coomassie Brilliant Blue G-250 stain before imaging on a Chemidoc^TM^ MP Imaging system (Bio-Rad Laboratories). pI values were calculated based on the length that the protein traveled through the IPG strip.

## 3. Results and Discussion

### 3.1. Polyacrylamide Gel Electrophoresis

**Figure 3:**
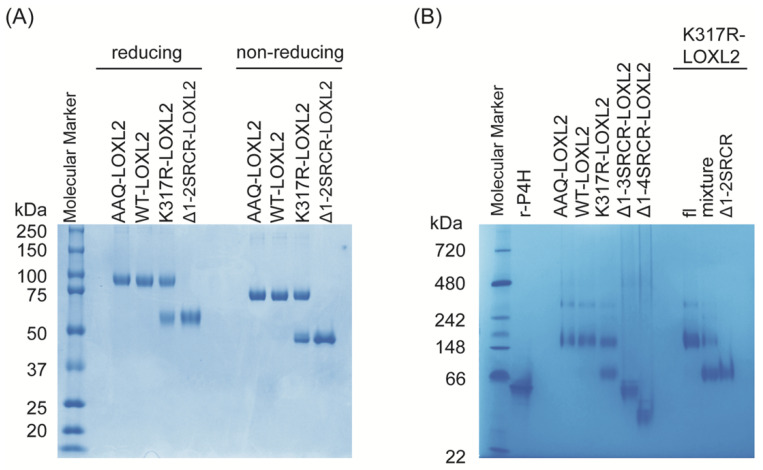
Polyacrylamide gel electrophoresis (PAGE) of LOXL2s. (**A**) SDS-PAGE under denaturing conditions in the presence and absence of reductant (β-mercaptoethanol). (**B**) Native-PAGE. AAQ: R315A/R316A/K317Q mutant form of LOXL2; WT: wild type; K317R-LOXL2 was isolated as a mixture of fl-LOXL2 and Δ1-2SRCR-LOXL2; Δ1-2SRCR-LOXL2 was isolated from the mixture by FPLC; r-P4H: recombinant *Bacillus anthracis* prolyl-4-hydroxylase as a molecular standard (homodimer, 49.2 kDa) [22].

Intermolecular association of two molecules of Zn^2+^-bound precursor Δ1-2SRCR-LOXL2 [19] is mediated mainly through SRCR4 domains via van der Waals interactions (3.3–4.0 Å) [27], as shown in Figure 2F. Although it was stated that the precursor Δ1-2SRCR-LOXL2 was monomeric in solution, no experimental data supporting this statement was provided [19]. In this study, both fl-LOXL2 and Δ1-2SRCR-LOXL2 in mature forms were purified to homogeneity (Figure 3A,B). fl-LOXL2 and Δ1-2SRCR-LOXL2 were subjected to polyacrylamide gel electrophoresis (PAGE) under both reducing and non-reducing conditions (Figure 3A). In the presence of a reducing reagent (all disulfide bonds are reduced), the molecular weights of fl-LOXL2 and Δ1-2SRCR-LOXL2 were ~95-kDa and ~60-kDa, respectively, which are in good agreement with the predicted values for monomers. In the absence of a reducing agent, both proteins are more compact and migrated as ~20-kDa smaller in size than when a reducing agent was added (Figure 3A) [28].

When these protein samples were subjected to native PAGE, Δ1-2SRCR-LOXL2 migrated (Figure 3B) similarly to the molecular weight under denaturing and reducing conditions (Figure 3A), suggesting that Δ1-2SRCR-LOXL2 is a monomer. Δ1-3SRCR- and Δ1-4SRCR-LOXL2s (Figure 3B) that lack the first three and all of the four SRCR domains also migrated to the expected molecular weights (42.7-kDa, 29.6-kDa) that were previously determined by mass spectrometry [21]. On the other hand, fl-LOXL2s of the wild-type (WT-LOXL2), R314A/R316A/K317Q-LOXL2 (AAQ-LOXL2, a PACE4 cleavage site null mutant) [4] and K317R-LOXL2 were detected as a single band with molecular weight of ~175-kDa but no protein band corresponding to the monomer, expected to be ~75-kDa under non-reducing conditions (Figure 3A), was detected. In addition to the ~175-kDa band, a faint band with a much higher molecular weight (~340-kDa) was detected in all three lanes containing fl-LOXL2 (AAQ-, WT- and K317R-LOXL2s) (Figure 3B). These results suggest that fl-LOXL2 exists predominantly as dimer and also as tetramer to a lesser extent. Since we did not detect dimers or other oligomers for Δ1-2SRCR-, Δ1-3SRCR-, and Δ1-4SRCR-LOXL2s, it is evident that one or both of the first two SRCR domains is/are essential for oligomerization of fl-LOXL2.

### 3.2. Molecular Weight Determination by SEC-MALS

**Figure 4:**
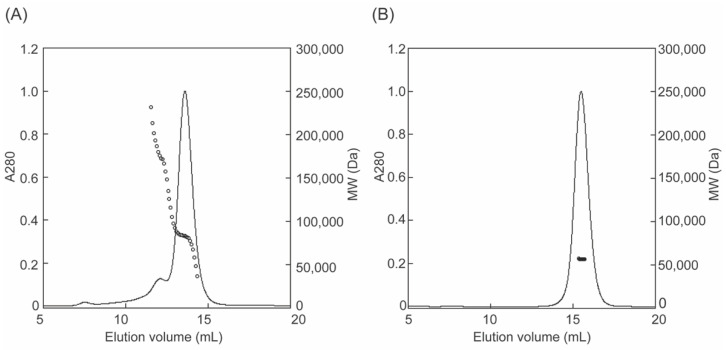
Molecular weight (MW) determination of LOXL2 by SEC-MALS (**A**) fl-LOXL2 (**B**) Δ1-2SRCR-LOXL2; for clarity only every 10th measurement of MW is plotted.

The molecular masses of fl-LOXL2 and Δ1-2SRCR-LOXL2 were determined by size exclusion chromatography coupled with multi angle light scattering (SEC-MALS) (Figure 4). At concentrations from 0.08 to 1.6 μM, fl-LOXL2 exists predominantly as the monomer with molecular weight of ~83-kDa at the elution volume of 13.6 mL but a small amount of dimer with molecular weight of ~176-kDa at the elution volume of 12.1 mL and a minor amount of tetramer (unable to calculate the molecular weight) at the elution value of 7.6 mL (Figure 4A). On the other hand, Δ1-2SRCR-LOXL2 elutes solely as a monomer with molecular weight of ~56-kDa at the elution volume of 15.5 mL (Figure 4B). The amounts of oligomers (dimer, tetramer) in Figure 4A in comparison to those in native acrylamide gel electrophoresis (Figure 3B) strongly indicates that fl-LOXL2 is predominantly a monomer but it is in an equilibrium of monomer-dimer-tetramer.

### 3.3. The Oligomeric State of fl-LOXL2

**Figure 5:**
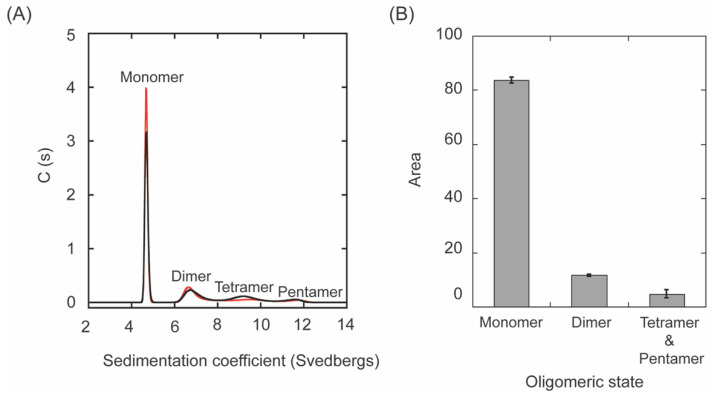
The oligomeric state of fl-LOXL2. (**A**) A representative run of SV-AUC with 0.88 mg/mL of fl-LOXL2. (**B**) A digitized graph showing the percentage of monomer, dimer, tetramer and pentamer of fl-LOXL2.

Since some oligomers (e.g., mostly dimers and tetramers in a lesser amount) were observed for fl-LOXL2 in the native PAGE and SEC-MALS (Figure 3B, Figure 4 and Figure 5), oligomerization of fl-LOXL2 was observed by sedimentation velocity–analytical ultracentrifugation (SV-AUC) at 20 °C with rotor speed at 40,000 RPM. Three different concentrations (0.88 mg/mL, 0.30 mg/mL and 0.088 mg/mL) were run to verify the system was not interacting on the timescale of the experiment. The weight average sedimentation coefficient showed no obvious trend with concentration (Figures S1 and S2) of fl-LOXL2 indicating a non-interacting system. A disperse distribution of four populations at monomer (~4.7 s), dimer (~6.9 s), tetramer (~9.3 s), and pentamer (~11.5 s) were observed (Figure 5A). Distribution integrations calculated were 83.6 ± 1.1% of monomer, 11.6 ± 0.3% of dimer and 4.7 ± 1.5% of tetramer and pentamer together (Figure 5B).

The shape of a molecule can be assessed from SV-AUC data by examining the frictional ratio, f/f_0_, which can be thought of as a measure of asymmetry. F/F_0_ is equal to s_max_/s*, the ratio of the maximum theoretical sedimentation coefficient (that of a perfect sphere) to the observed sedimentation coefficient. If we use ~86.2-kDa for the monomer of LOXL2, the maximum of sedimentation coefficient can be calculated as ~7.0 s (s: Svedberg unit, 10^−13^ s) by using equation: $s_{\max}=0.00361M^{\frac{2}{3}}$ (s_max_: the maximum theoretical sedimentation coefficient, M = molecular weight) [29,30]. The observed sedimentation coefficient is ~4.7 s, that will make f/f_0_ = s_max_/s* = 7.0 s/4.7 s = 1.5. This indicates that fl-LOXL2 is a moderately asymmetrical protein (Table 1). From these data we can also calculate a Stokes radius (radius of the equivalent sphere that sediments at the same rate) of 4.5 nm.

### 3.4. Hydrodynamic Radii (R_h_) of fl- and Δ1-2SRCR-LOXL2

**Figure 6:**
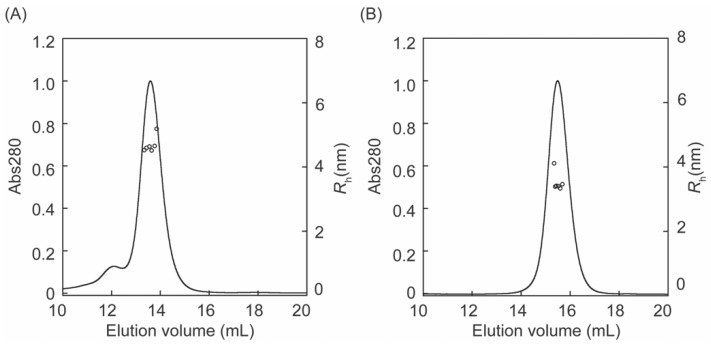
Hydrodynamic radii *(R*_h_) of fl-LOXL2: (**A**) Δ1-2SRCR-LOXL2 (**B**) calculated from SEC-MALS analysis.

Hydrodynamic radii (*R*_h_) of fl-LOXL2 and Δ1-2SRCR-LOXL2 were calculated from the “on-line” dynamic light scattering measurement (Figure 6, Table 1) and the dynamic light scattering signal was analyzed by the method of cumulants [24]. The *R*_h_ value of fl-LOXL2 (4.58 ± 0.05 nm) is comparable to that (4.98 ± 0.08 nm) of aldolase, a globular protein that has been routinely used as the standard [31,32]. However, the molecular weight of aldolase is 158-kDa that is larger than the monomer of fl-LOXL2 (~86-kDa). On the other hand, the *R*_h_ value of Δ1-2SRCR-LOXL2 (3.40 ± 0.15 nm) is in good agreement with those (3.40–3.69 nm) of bovine serum albumin and human serum albumin which both have similar mass (66.5-kDa) [31] to that of Δ1-2SRCR-LOXL2 (~66 kDa). These results suggest that fl-LOXL2 is a moderately elongated protein but Δ1-2SRCR-LOXL2 is an approximately globular protein. While the difference (~1.2 nm) between *R*_h_ values of fl-LOXL2 and Δ1-2SRCR-LOXL2 is significant, it is still considerably smaller than the difference in molecular weights (~20-kDa). Therefore, the difference cannot account for a fully extended conformation of the first two SRCR domains of fl-LOXL2 (SRCR1 and SRCR2).

### 3.5. Structure Prediction of fl-LOXL2

**Figure 7:**
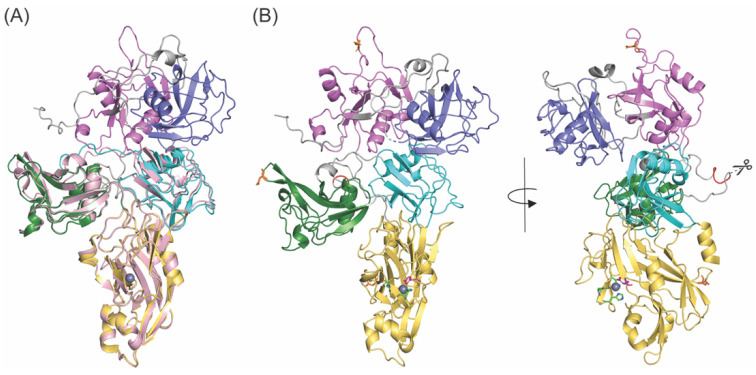
Structure prediction of fl-LOXL2. (**A**) Overlay of 3D structure of fl-LOXL2 predicted by AlphaFold ver 2 (SRCR1 in slate, SRCR2 in purple, SRCR3, SRCR4, amine oxidase domain in lightpink) and X-ray crystal structure of Δ1-2SRCR-LOXL2 (SRCR3 in cyan, SRCR4 in green, amine oxidase domain in yellow). Zn^2+^ occupying the Cu^2+^-binding site is shown as a gray sphere. (**B**) 3D structure of fl-LOXL2 predicted by AlphaFold ver 2 (SRCR 1 in slate, SRCR in purple, SRCR3 in cyan, SRCR4 in green, amine oxidase domain in yellow) in two angles. The PACE4 cleavage site (^314^Arg-^315^Phe-^316^Arg-^317^Lys-↓^318^Ala) is highlighted in red. The *N*-glycosylation sites (Asn288, Asn455, Asn644) are in orange stick. Peptides connecting domains are in gray.

The overlaid image (Figure 7A) of the 3D structure of fl-LOXL2 (precursor form) that was predicted by AlphaFold 2 (AF-Q9Y4K0-F1-model_vi.pdb) [20] and X-ray crystal structure of Δ1-2SRCR-LOXL2 (precursor form) were generated by PyMOL [33]. The third and fourth SRCR domains and the C-terminal amine oxidase domain of the 3D modeled structure were superimposable with those of the X-ray structure, where the root mean square deviation (RMSD) for the overlay was calculated as 0.490 Å. The first and second SRCR domains (in slate and in purple, respectively) of the 3D modeled structure have domain–domain interactions with the third SRCR domain (in cyan) but not with the fourth SRCR domain (in green) (Figure 7B, left). As seen in the crystal structure of Δ1-2SRCR-LOXL2, there is no direct interaction between the third and fourth SRCR domains of the 3D predicted structure. The PACE4 cleavage site (^314^Arg-^315^Phe-^316^Arg-^317^Lys-↓^318^Ala) [4] is in the loop region between the second and the third SRCR domains and solvent-exposed (Figure 7B, right). The three *N*-glycosylation sites (Asn288, Asn455, Asn644) [34] are all solvent-exposed as well. The *R*_h_ value of the 3D predicted structure of fl-LOXL2 was calculated as 4.48 nm by HullRad [31] that is within a reasonable margin of error of the *R*_h_ value (4.58 ± 0.05 nm) calculated from our mature fl-LOXL2 in solution by SEC-MALS (Table 1). These results indicate that the overall domain organizations of the 3D modeled precursor fl-LOXL2 are closely related to the mature fl-LOXL2 in solution.

During the course of our study, *R*_h_ values of 5.6–6.6 nm for a fl-LOXL2 were calculated from SEC-MALS and single-particle electron microscopy (EM), respectively [5]. In that study, a recombinant fl-LOXL2 was purified from the conditioned media of Chinese hamster ovary cells stably transfected with an expression construct of C-terminally His-tagged fl-LOXL2. The EM images after negative staining indicated that the four SRCR domains of the fl-LOXL2 are fully extended and there was no sign of domain–domain interactions. The *R*_h_ values determined for the fl-LOXL2 are 1–2 nm (10–20 Å) larger than those calculated for both fl-LOXL2 and the 3D predicted structure (AlphaFold V2) that have been previously discussed in this study (Table 1). As mentioned earlier, in our hands, the difference observed in *R*_h_ values (1.18 ± 0.02 nm, or 11.8 ± 0.2 Å) between fl-LOXL2 and Δ1-2SRCR-LOXL2 (mature forms) are in good agreement with the difference in *R*_h_ values calculated (1.36 nm, 13.6 Å) between the 3D-predicted structure of fl-LOXL2 and the X-ray crystal structure of the monomer of Δ1-2SRCR-LOXL2 (precursor forms) by HullRad [31]. Our results strongly suggest that the overall structures of both fl-LOXL2 and Δ1-2SRCR-LOXL2 (mature forms) in solution are related to those of 3D-predicted fl-LOXL2 and X-ray crystal structure of Δ1-2SRCR-LOXL2 (precursor forms). The difference in *R*_h_ value determined by EM may be due to the acidic environment (pH 4.7) for staining.

### 3.6. The Isoelectric Point (pI) of LOXL2

The isoelectric points (pIs) of a series of recombinant LOXL2s were determined by isoelectric focusing polyacrylamide gel electrophoresis (IEF-PAGE) (Table 2). The experimentally determined pIs are in good agreement with values predicted by Isoelectric Compute pI/MW [35] and Point Calculator 2.0 [36]. A slight smearing of fl-LOXL2 was detected and two major spots gave a pI of 5.5–5.9. This is most likely due to the heterogeneity of N-glycoconjugates (31 varieties at Asn288) [34]. Although N-glycoconjugates at Asn644 also possess 34 varieties [34], they did not have significant influence on pI values for all forms of recombinant LOXL2 in this study. Overall, LOXL2 is a weakly acidic protein. A surface electrostatic potential map generated by PyMOL with APBS Electrostatistics plug-in [37] shows a large acidic patch expanding from the amine oxidase domain through the SRCR3 domain to the SRCR1 domain in one face of fl-LOXL2 (Figure S3A). On the other hand, there is a basic patch present in the SRCR2 domain and another acidic patch in the amine oxidase domain on the other face of fl-LOXL2 (Figure S3B). The estimated pI value of the SRCR2 domain is 8.8–9.6 (Table 3), which is over 3 pH unit more basic than other three SRCR domains. Further study is necessary to define the biological and physiological significance of the basicity of the SRCR2 domain.

A significant amount of dimerization of LOXL2 was only observed with fl-LOXL2 at concentrations ≥0.88 mg/mL (Figure 3 and Figure 5). As the dimerization only occurred within fl-LOXL2, it is most likely that the dimerization involves one or both of the first two SRCR domains. Two docking programs, PatchDock and ZDOCK were used to generate possible modes of dimerization [38,39,40]. While the most common pattern seen was interaction between SRCR1 of monomer and SRCR2 of the other monomer (an example is shown in Figure S4), some models that were generated showed interactions between the first SRCR domain and the catalytic domain. More study is necessary, such as H/D exchange mass spectrometry, site-directed mutagenesis, or deletion of the binding motif to elucidate the nature of the dimerization of fl-LOXL2.

## 4. Conclusions

In solution, fl-LOXL2 exists predominantly as a monomer, but it can be in equilibrium with dimer–tetramer–pentamer when the protein concentration is ≥0.88 mg/mL (monomer: 83.6%; dimer: 11.6%; tetramer + pentamer: 4.7%). On the other hand, Δ1-2SRCR-, Δ1-3SRCR-, and Δ1-4SRCR-LOXL2s exists exclusively as a monomer. These results suggest that the dimerization of fl-LOXL2 involves one or both of the first two SRCR domains. The pI value of the SRCR2 domain is basic (~9.2), in contrast to the rest of the SRCR domains and the catalytic domain of LOXL2 (5~6). This basicity of the SRCR2 domain potentially mediates protein–protein interaction in the ECM or cell-surface through ionic interactions. The higher affinity of fl-LOXL2 to the ECM that we previously observed [4] could partly be attributed to the basicity of the SRCR2 domain. LOXL2 has been shown to oxidatively deaminate Lys residue(s) of the extracellular domain of PDGFRβ to an aldehyde [6]. The pI value of the extracellular domain of PDGFRβ is predicted to be 4.83 and 4.71 by Isoelectric Compute pI/MW [35] and Point Calculator 2.0 [36], respectively. It is possible that the interaction of LOXL2 and PDGFRβ is facilitated by the SRCR2 domain. The *R*_h_ values determined for fl-LOXL2 and Δ1-2SRCR-LOXL2 in solution strongly suggest that the overall structures of both fl-LOXL2 and Δ1-2SRCR-LOXL2 in solution are closely related to those of 3D-predicted fl-LOXL2 and X-ray crystal structure of Δ1-2SRCR-LOXL2, and the LTQ cofactor biogenesis does not have a significant effect on the overall structure of LOXL2.

## Figures and Tables

**Table 1:** A comparison of biophysical data of fl-LOXL2 and Δ1-2SRCR-LOXL2.

LOXL2	pH	Methods	MW (kDa)	*R*_g_ (nm)	*R*_h_ (nm)	10^13^ s (sec)	f/f_0_
fl- ^(a)^	8.0	AUC/SEC-MALS	83/91 ^(f)^	−	4.58 ± 0.05	4.7	1.49
Δ1-2SRCR- ^(a)^	8.0	AUC/SEC-MALS	57.3	−	3.40 ± 0.15	−	−
fl- ^(b)^	−	Computation ^(e)^	86.7	3.31	4.48	4.73	1.54
Δ1-2SRCR- ^(c)^	7.6	Computation ^(e)^	49.6	2.41	3.12	3.94	1.29
fl- ^(d)^	7.4	AUC/SEC-MALS	92.6	−	5.6	5.15	1.44
fl- ^(d)^	4.7	TEM	93	4.7	6.6	5.52	−

(a) Purified recombinant proteins used in this study. (b) 3D structure predicted by AlphaFold V2 [20]. (c) The monomer part of the X-ray crystal structure (PDB:5ZE3) without water molecules. (d) Purified recombinant protein [4]. (e) Computational calculation by HullRad [31]. (f) Including *N*-glycans.

**Table 2:** Isoelectric points (pIs) of the series of recombinant LOXL2s in this study.

LOXL2	pI (Computed pI ^(a^^,b)^)
fl-LOXL2	5.5–5.9 (5.7, 6.0)
Δ1-2SRCR-LOXL2	5.4 (5.9, 5.9)
Δ1-3SRCR-LOXL2	5.9 (5.3, 5.1)
Δ1-4SRCR-LOXL2	5.1 (5.1, 4.9)

(a) Compute pI/Mw [36], (b) Isoelectric Point Calculator 2.0 [37].

**Table 3:** Isoelectric points (pIs) of SRCR domains of fl-LOXL2.

SRCR Domain of LOXL2	pI (Computed ^(a^^,b)^)
SRCR1	5.4, 5.2
SRCR2	9.6, 8.8
SRCR3	5.1, 5.0
SRCR4	5.9, 5.6

(a) Compute pI/Mw [36], (b) Isoelectric Point Calculator 2.0 [37].

## Data Availability

The data within this article are available from the authors upon request but may require data transfer agreement.

## Supplementary Materials

The following are available online at https://www.mdpi.com/article/10.3390/biom11121846/s1, Figure S1: Weight average sedimentation coefficient with concentration of fl-LOXL2, Figure S2: Data, fit, and residuals plot for a representative SV-AUC run of fl-LOXL2 at 0.88 mg/mL, Figure S3: Electrostatic potential on the surface of 3D-predicted LOXL2, Figure S4: A predicted dimer of fl-LOXL2 generated by ZDOCK.
