# Supplementary material for: Oligomeric States and Hydrodynamic Properties of Lysyl Oxidase-Like 2
Source: Biomolecules. 2021 Dec 8;11(12):1846. doi: 10.3390/biom11121846 (PMC8699698; doi:10.3390/biom11121846)
Supplement: Supplementary file 1 [file biomolecules-11-01846-s001.zip › biomolecules-1471216-supplementary.pdf]

Supporting Materials

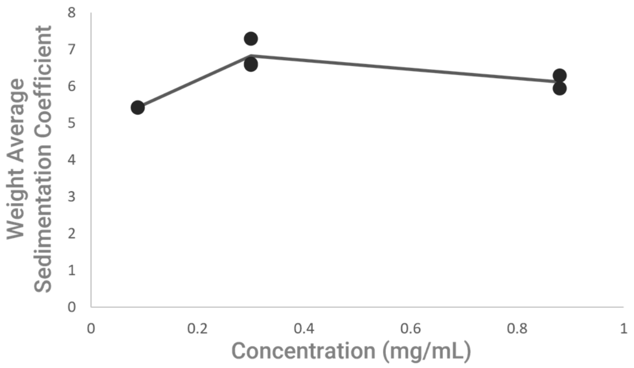

Figure S1: Weight average sedimentation coefficient with concentration of fl-LOXL-2. The weight average sedimentation coefficient is a reduction of the sedimentation coefficient distribution to a single descriptive number - the average sedimentation coefficient weighted by signal. A trend of increasing size (and therefore increasing weight averaged sedimentation coefficient) is indicative of a system interacting via reversible self-association, while a flat trend as observed above is an indication of a non-interacting system.

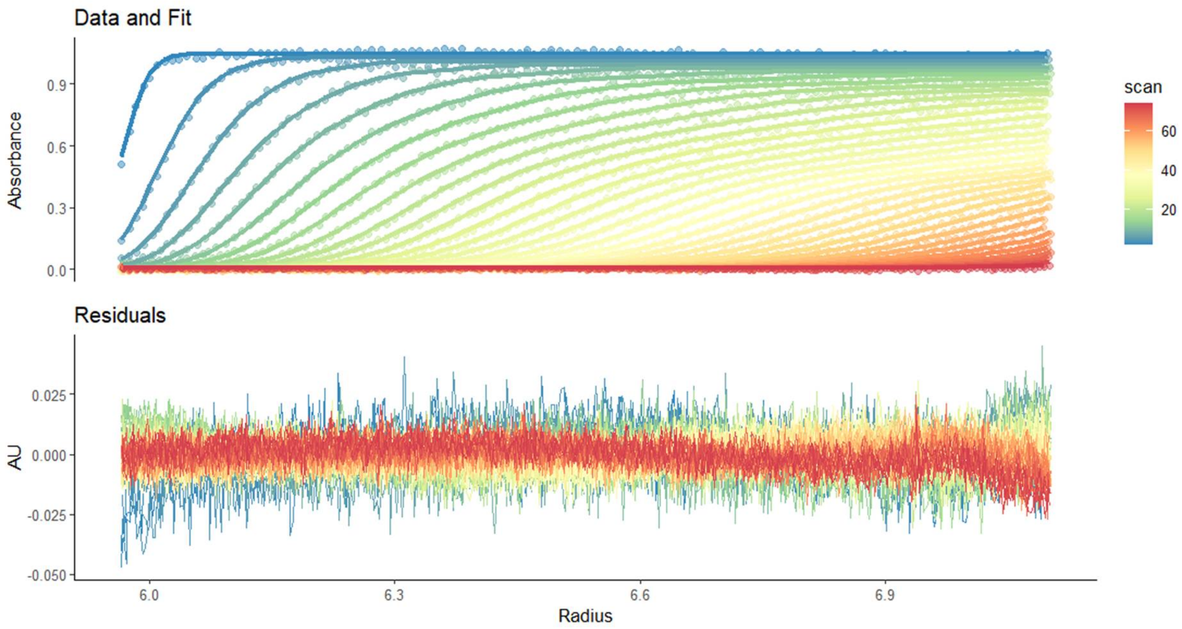

Figure S2: Data, fit, and residuals plot for a representative SV-AUC run of fl-LOXL2 at 0.88 mg/mL.

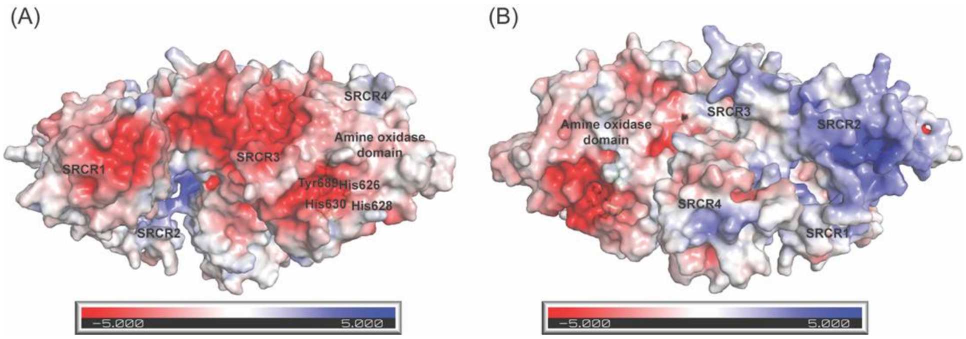

Figure S3. Electrostatic potential on the surface of 3D-predicted LOXL2. (A) Acidic patches spanning SRCR1 domain through SRCR3 domain and the C-terminal amine oxidase domain. **(B)** A horizontally flipped image of (A). On the left is the backside of the active site. A basic groove exists in SRCR2 domain.

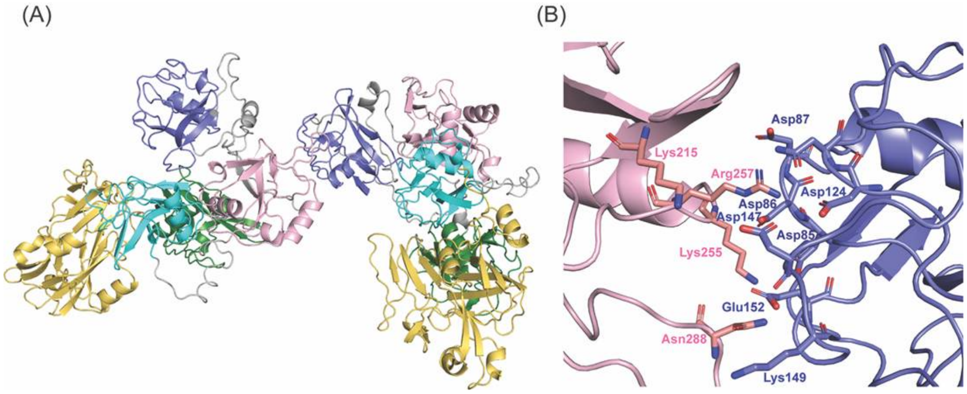

Figure S4. A predicted dimer of fl-LOXL2 generated by ZDOCK. (A) Molecular docking of the monomer of fl-LOXL2 without the signal peptide, residues 1-25, where interactions are limited to the first two SRCR domains. **(B)** The interactions between the SRCR1 domain of monomer 1 and the SRCR2 domain of monomer 2 are mostly ionic and between acidic residues of SRCR1 and basic residues of SRCR2. These interactions are limited to small loops on both domains and may explain why fl-LOXL2 is primarily monomeric.
